# Supplementary material for: A Facile Synthesis of (PIM-Polyimide)-(6FDA-Durene-Polyimide) Copolymer as Novel Polymer Membranes for CO2 Separation
Source: Membranes (Basel). 2019 Aug 31;9(9):113. doi: 10.3390/membranes9090113 (PMC6780089; doi:10.3390/membranes9090113)
Supplement: Supplementary file 1 [file membranes-09-00113-s001.pdf]

membranes

Article

Supplementary Materials: A Facile Synthesis of (PIM-Polyimide)-(6FDA-Durene-Polyimide) Copolymer as Novel Polymer Membranes for CO_2 Separation

Iqubal Hossain [1,2], Abu Zafar Al Munsur [1,2] and Tae-Hyun Kim [1,2,*]

1 Organic Material Synthesis Laboratory, Department of Chemistry, Incheon National University, Incheon, 22012, Korea.
2 Research Institute of Basic Sciences, Incheon National University, Incheon, 22012, Korea.
* Correspondance: tkim@inu.ac.kr (T.-H. Kim); Tel. +82-32-8358232.

S-1. Materials

4,4′-(Hexafluoroisopropylidene) di-phthalic anhydride (6FDA), 2,3,5,6-tetramethyl benzene-1,4-diamine (durene), and 4,5-dichloro phthalonitrile (98%), were purchased from Tokyo Chemical Industry (TCI) Co. Ltd. (Tokyo, Japan) and were used as obtained. 5,5′,6,6′-Tetrahydroxy-3,3,3′,3′-tetramethyl-1,10-spirobisindane (97%) was obtained from Alfa Aesar. Acetic anhydride, toluene, and triethylamine were obtained from Sigma Aldrich. Methanol, ethanol, dimethylformamide, dimethylacetamide, potassium carbonate, and potassium hydroxide were purchased from DaeJung Chemicals & Metals Co. Ltd. in South Korea. 6FDA, durene, and K_2CO_3 were dried under a vacuum at 60 °C for 24 h prior to use. Anhydride monomer of PIM (**An**) was synthesized following the literature method [22]. All chemicals, unless otherwise noted, were obtained from commercial sources and were used as received.

S-2. Characterization and Measurements

The ^{1}H NMR spectra were obtained on an Agilent 400-MR (400 MHz) instrument using d_6-DMSO or $CDCl_3$ as a reference or an internal deuterium lock.

The attenuated total reflection Fourier transform infrared (ATR-FTIR) spectra were recorded using a Bruker Vertex 80v Hyperion 2000 ATR-FTIR spectrometer.

Molar masses were determined by gel permeation chromatography (GPC) using two PL Gel 30 cm × 5 μm mixed C columns at 30 °C running in DMF and calibrated against polystyrene (M_n = 600×10^6 gmol^{-1}) standards using a Knauer refractive index detector.

The thermal stability of the membranes was analyzed by thermogravimetric analysis (TGA) measurements conducted on a Shimadzu TGA-2950 instrument at a heating rate of 10 °C min^{-1} under a nitrogen flow.

The tensile properties were measured on a Shimadzu EZ-TEST E2-L instrument benchtop tensile tester using a crosshead speed of 5 mm.min^{-1} at 25 °C under 50% relative humidity. The engineering stress was calculated from the initial cross-sectional area of the sample and Young's modulus (E) was determined from the initial slope of the stress-strain curve. The membrane samples were cut into rectangular shapes 40 mm × 10 mm (total) and 20 mm × 10 mm (test area) in size.

The densities of the membranes (g.cm^{-3}) were determined experimentally using a top-loading electronic Mettler Toledo balance (XP205, Mettler-Toledo, Switzerland) coupled with a density kit based on the Archimedes principle. The samples were weighed in air and in a known-density liquid, high-purity heptane. The measurements were performed at room temperature using the buoyancy method, and the density was calculated as follows:

$$\rho_{polymer} = \frac{W_0}{W_0 - W_1} \cdot \rho_{liquid} \quad \text{(S1)}$$

where W_0 and W_1 are the membrane weights in air and in heptane respectively. Heptane sorption of the membranes was not considered due to their extremely low absorption properties.

The X-ray diffraction patterns of the membranes were measured using a Rigaku DMAX-2200H diffractometer operated at a scanning rate of 4° min^{-1} in a 2θ range from 5° to 30° with Cu Kα1 X-ray radiation (λ = 0.1540598). The *d*-spacings were calculated using Bragg's law ($d = \lambda/2\sin\theta$).

Tapping-mode AFM was conducted using a Bruker MultiMode instrument. A silicone cantilever with an end radius of <10 nm and a force constant of 40 Nm^{-1} (NCHR, nanosensors, f=300 kHz) was used to image the samples at an ambient temperature.

S-3. Gas Permeation Procedure

Permeation measurements of pure gas were taken using a high-vacuum time-lag measurement unit based on a constant-volume/variable-pressure method. All of the experiments were performed at a feed pressure of 2 bar (except for the pressure effect experiments which were carried out in the range of 100 mbar to 2 bar feed pressure) and a feed temperature of 30 °C. Before taking these measurements, both the feed and the permeate sides were thoroughly evacuated to below 10^{-5} Torr (1.33×10^{-8} bar) until the readout showed zero values for the removal of any residual gases. The downstream volume was calibrated using a Kapton membrane and was found to be 50 cm^3. The upstream and downstream pressures were measured using a Baraton transducer (MKS; Model No. 626B02TBE) with a full scale of 10,000 and 2 Torr (13.3 and 2.7×10^{-3} bar), respectively. The pressure on the permeate side was recorded as a function of time using a pressure transducer and passed to a desktop computer through a shielded data cable. The permeability coefficient was determined from the linear slope of the downstream pressure versus a time plot (d*p*/d*t*) according to the following equation:

$$\boldsymbol{P} = \frac{\mathbf{273}}{\mathbf{76}} \times \frac{\boldsymbol{Vl}}{\boldsymbol{ATp_0}} \times \frac{\boldsymbol{dp}}{\boldsymbol{dt}} \quad \text{(S2)}$$

where, *P* is the permeability expressed in Barrer (1 Barrer = 10^{-10} [cm^3 (STP) $cm.cm^{-2}.s^{-1}.cm^{-1}.Hg^{-1}$], *V* ($cm^3$) is the downstream volume, *l* (cm) is the membrane thickness, *A* (cm^2) is the effective area of the membrane, *T* (K) is the measurement temperature, p_o (Torr) is the pressure of the feed gas in the upstream chamber, and *dp*/*dt* is the rate of the pressure change under a steady state. For each gas, the permeation tests were repeated more than three times, and the standard deviation from the mean values of the permeabilities was within ca. ± 3%. Sample-to-sample reproducibility was high and within ± 3%. The effective membrane areas were 15.9 cm^2. The ideal permselectivity, $\alpha_{A/B}$, of the membrane for a pair of gases (A and B) is defined as the ratio of the individual gas permeability coefficients:

$$\boldsymbol{\alpha_{A/B}} = \frac{\boldsymbol{P_A}}{\boldsymbol{P_B}} \quad \text{(S3)}$$

The diffusivity and solubility were obtained from the time-lag (θ) value according to the equations:

$$\boldsymbol{D} = \frac{\boldsymbol{l^2}}{\boldsymbol{6\theta}} \quad \text{(S4)}$$

$$\boldsymbol{S} = \frac{\boldsymbol{P}}{\boldsymbol{D}} \quad \text{(S5)}$$

where, *D* (cm^2 s^{-1}) is the diffusivity coefficient, *l* is the membrane thickness (cm) and θ is the time lag (s), as obtained from the intercept of the linear steady-state part of the downstream pressure versus a time plot. The solubility, *S*, was calculated from Equation. (S**5**) with the permeability and diffusivity obtained from Equation. (S**3**) and (S**4**).

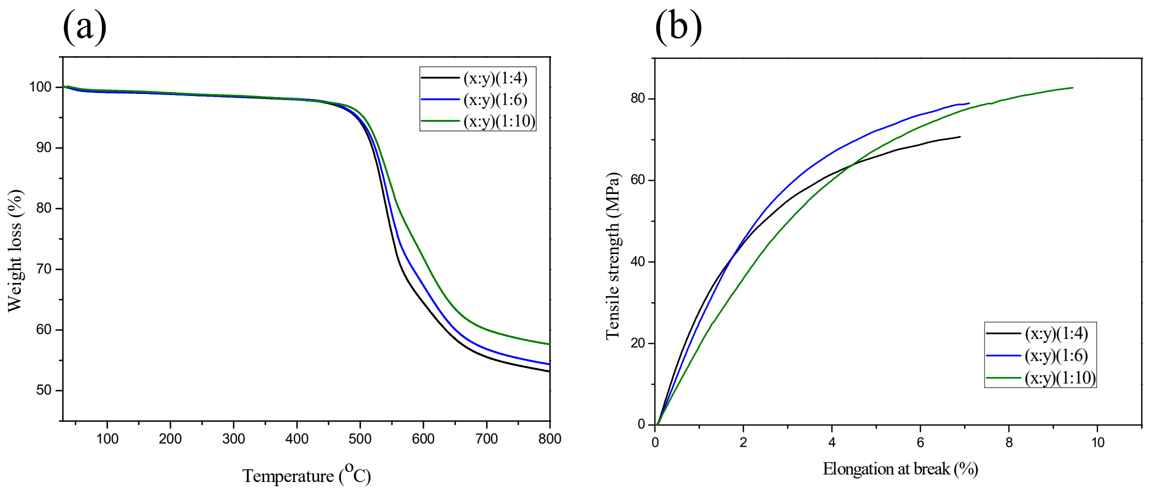

Figure S1. TGA graph (**a**) and S-S curve (**b**) of the copolymer [(PIM-PI)$_x$-(6FDA-durene-PI)$_y$] membranes.

© 2019 by the authors. Submitted for possible open access publication under the terms and conditions of the Creative Commons Attribution (CC BY) license (http://creativecommons.org/licenses/by/4.0/).
